# Human Papillomavirus Vaccination Over Time in Heterosexual and Sexual Minority Adults in the United States

**DOI:** 10.1089/heq.2021.0058

**Published:** 2022-04-14

**Authors:** Chloe Chan, Amandeep K. Mann, Danny Lee, Lexi Nutkiewicz, Kathleen T. Watson, Daniel S. Kapp, Juno Obedin-Maliver

**Affiliations:** ^1^ Palo Alto Medical Foundation Research Institute, Palo Alto, California, USA.; ^2^ Department of Psychiatry, Stanford University School of Medicine, Stanford, California, USA.; ^3^ Department of Radiation Oncology, Stanford University School of Medicine, Stanford, California, USA.; ^4^ Department of Epidemiology and Population Health, Stanford University School of Medicine, Stanford, California, USA.

**Keywords:** human papillomavirus vaccination initiation, human papillomavirus vaccination, human papillomavirus, NHANES, sexual minorities, factors associated with HPV vaccination, LGBT Persons

## Abstract

**Purpose:**

We proposed to identify the factors that determine the trends in human papillomavirus (HPV) vaccination initiation and completion among heterosexual and sexual minority adults.

**Methods:**

Using National Health and Nutrition Examination Survey database from 2007 to 2016, we performed chi-squared tests and multivariate logistic regression analysis.

**Results:**

Heterosexual females initiated vaccination at 23.5% compared with sexual minority females at 34.6% (*p*<0.001). Although heterosexual males also had a lower vaccination initiation than sexual minority males (7.7% vs. 15.5%; *p*=0.12), their completion rate appeared higher (38% vs. 17%; *p*=0.14).

**Conclusion:**

Interventions are needed to enhance support for completion rates of HPV vaccine among sexual minority individuals.

## Introduction

Sexual minorities have been shown to have poorer access to health care, particularly in preventative medicine and differences associated with human papillomavirus (HPV)-related outcomes and care provision such as Pap smear utilization, a marker for the development of cervical and anal cancer.^1–4^ Prior HPV vaccination studies did not explicitly include or assess sexual minority communities or the setting of health care received.^5^ In this study, we examined the trends of HPV vaccination and health care access patterns in sexual minority and heterosexual adults.

## Methods

National Health and Nutrition Examination Survey (NHANES) database is an ongoing study conducted by the Centers for Disease Control and Prevention that collects information, in a 2-year cycle, on health and nutritional status of adults and children in the United States.^6^ Our cross-sectional study consisted of participants of ages 20–36 years with self-reported information on demographics, health care characteristics, self-identified sexual orientation, and HPV vaccination from 2007 to 2016. The upper age limit was restricted to 36 years in our study as these individuals would have been 26 years old in 2006 when the earliest form of the HPV vaccine was Food and Drug Administration approved.

Age was dichotomized using the median value, 26 years. Participants self-reported their sexual orientation as either “heterosexual,” “homosexual,” “bisexual,” “something else,” “not sure,” “refused,” or “don't know.” Sexual minority was defined as homosexual and bisexual. Individuals who received at least one dose of the vaccine were defined as having “initiated” the vaccine, whereas those who received three doses were defined as having “completed” the vaccine series. Participants were also surveyed regarding the setting where they received health care. Listwise deletion was used to handle missing data or responses of “refused” or “don't know” on demographics, health care characteristics, self-identified sexual orientation, and HPV vaccination from our analysis.

Using SAS Enterprise Guide version 7.1 (SAS Institute Inc., Cary, NC), SAS PROC SURVEY procedures were used to conduct chi-square tests and multivariate logistic regression models. We examined interaction terms of sexual minority with gender as well as with race in an additional multivariable logistic regression. However, the interaction showed insignificant findings. A trend test was used to analyze the change for HPV vaccination. We incorporated cluster, strata, and weighted variables to ensure that oversampling of any groups did not occur. A *p*-value of ≤0.05 was considered statistically significant. Since our study data contain no participant identifying information, it was institutional review board approval exempt.

## Results

Of 4115 adult participants with median age of 26 years (20–36), 60.3% had female gender classification and 39.7% had male gender classification. More than half identified as White (59.8%), followed by Black (12.8%), Hispanic (18.3%), and other race (9.1%). Majority of participants reported receiving their health care from the doctor's office or Health Maintenance Organization (HMO) (46.8%). Overall, 92% identified as heterosexual and 8% as sexual minority. The overall vaccination rate was 18.1%, with 17% in the heterosexual group versus 29.8% in the sexual minority group (*p*<0.001; Table 1). More specifically, 23.5% of heterosexual females initiated vaccination compared with 34.6% in sexual minority females (*p*<0.001).

**Table 1.** Human Papillomavirus Vaccination Initiation (≥1 Dose) Rates by Sexual Orientation in National Health and Nutrition Examination Survey (2007–2016)

Factors	Overall (***n***=4115), % (95% CI)	Heterosexuals^a,b^ (***n***=3785), % (95% CI)	Sexual minorities^a,c^ (***n***=330), % (95% CI)	***p*** ^d^
HPV vaccination initiation rate	18.1 (16.2–19.9)	17.0 (15.1–19.0)	29.8 (23.2–36.5)	**<0.001**
Age group				
26 years and younger	25.5 (22.8–28.1)	24.2 (21.3–27.1)	37.0 (29.0–45.0)	**0.01**
Older than 26 years	10.0 (8.3–11.8)	9.6 (8.0–11.2)	16.7 (7.0–26.5)	0.13
Gender^e^				
Female	24.6 (22.0–27.2)	23.5 (20.7–26.3)	34.6 (26.4–42.8)	**0.01**
Male	8.1 (6.1–10.1)	7.7 (5.7–9.8)	15.5 (5.6–25.3)	0.12
Race				
White	20.0 (17.4–22.6)	18.9 (16.1–21.8)	31.7 (22.6–40.8)	**0.01**
Black	19.1 (16.1–22.0)	18.0 (15.1–20.9)	29.3 (18.3–40.2)	**0.047**
Hispanic	12.3 (10.1–14.4)	11.7 (9.6–13.7)	22.6 (9.2–36.1)	0.12
Other^f^	15.6 (12.2–19.1)	14.5 (11.2–17.7)	27.8 (11.2–44.4)	0.12
Education				
Below high school	7.7 (5.4–10.0)	7.3 (5.2–9.5)	12.4 (2.2–22.5)	0.32
High school	13.8 (11.0–16.6)	12.7 (9.7–15.6)	24.3 (13.2–35.4)	0.07
Associates and some college	23.0 (20.4–25.6)	21.9 (19.2–24.6)	34.0 (24.7–43.2)	**0.02**
College graduate and above	18.9 (15.6–22.2)	17.8 (14.5–21.1)	37.5 (22.9–52.0)	**0.02**
Income				
High	18.6 (15.4–21.8)	17.4 (14.0–20.8)	36.7 (23.6–49.8)	**0.01**
Middle	19.6 (16.7–22.5)	18.7 (15.7–21.6)	30.7 (18.9–42.4)	0.06
Low	15.9 (13.5–18.4)	14.9 (12.6–17.3)	25.1 (14.9–35.2)	0.051
Type of insurance				
None	11.1 (9.0–13.3)	10.6 (8.4–12.7)	16.7 (9.0–24.3)	0.12
Private	20.5 (17.8–23.2)	19.1 (16.3–21.8)	39.6 (28.3–50.9)	**0.001**
Medicare	6.6 (0.0–16.3)	5.0 (0.0–14.8)	16.4 (0.0–48.5)	0.48
Medicaid	18.8 (14.6–22.9)	18.9 (14.4–23.4)	17.7 (7.8–27.7)	0.84
Other^g^	23.3 (16.8–29.8)	22.2 (14.9–29.4)	33.6 (10.0–57.2)	0.38
Setting of routine health care received				
No place	11.5 (9.2–13.7)	10.3 (8.1–12.5)	26.3 (13.9–38.7)	**0.01**
Clinic or health center	17.8 (14.5–21.0)	16.7 (13.5–20.0)	26.9 (14.3–39.5)	0.13
Doctor's office or HMO	21.2 (18.5–23.8)	20.2 (17.4–23.0)	35.0 (24.7–45.2)	**0.01**
Hospital emergency room	16.8 (10.8–22.8)	16.9 (10.2–23.6)	15.9 (1.1–30.7)	0.90
Other^h^	28.8 (19.8–37.8)	27.1 (17.6–36.7)	40.6 (7.6–73.5)	0.44
Time period^*^				
2007–2008	10.8 (4.6–16.9)	11.1 (4.5–17.8)	6.3 (0.0–18.4)	0.49
2009–2010	15.3 (11.1–19.5)	15.5 (11.0–20.0)	13.4 (3.8–23.0)	0.68
2011–2012	15.1 (11.3–18.9)	13.9 (10.0–17.8)	32.0 (18.6–45.3)	**0.01**
2013–2014	18.9 (15.3–22.4)	17.7 (14.2–21.2)	32.8 (21.8–43.7)	**0.003**
2015–2016	22.7 (19.0–26.3)	21.2 (17.2–25.2)	39.3 (23.3–55.3)	**0.04**

*Note:* Numbers do not add up to 100% because of subgroup analyses. Data reports human papillomavirus vaccination rate. Boldface indicates statistical significance (*p*<0.05).

^*^ Overall: *p*-trend <0.001; heterosexuals: *p*-trend=0.004; sexual minority: *p*-trend=0.001.

^a^ Participants were asked in the survey, “Do you think of yourself as…” with options given as heterosexual or straight, “homosexual, bisexual, something else, not sure, refused, and don't know” (https://www.cdc.gov/nchs/nhanes).

^b^ Male heterosexuals=1549; female heterosexuals=2236.

^c^ Male homosexuals=49; male bisexuals=25; female homosexuals=45; female bisexuals=211.

^d^ Chi-square test used to test for association between sexual behavior and HPV vaccination by each category.

^e^ Gender options in NHANES are presented as female or male only.

^f^ Other race includes multirace and any other race not specified in the NHANES database.

^g^ Other insurance includes Medi-Gap, State Children's Health Insurance Program, military health insurance, Indian health service, state-sponsored health plan, and other government insurance.

^h^ Other setting of health care includes hospital outpatient department and other place not specified in the NHANES database.

CI, confidence interval; HMO, Health Maintenance Organization; HPV, human papillomavirus; NHANES, National Health and Nutrition Examination Survey.

Those who received routine health care in the doctor's office or HMO setting had an overall vaccination rate of 21.2%; of this group, heterosexual adults were less likely to receive vaccination at 20.2% versus 35% in sexual minority adults (*p*=0.01). Our data suggest that heterosexual males initiated vaccination at a lower rate than sexual minority males (7.7%, 95% confidence interval [CI]: 5.7–9.8% vs. 15.5%, 95% CI: 5.6–25.3%; *p*=0.12). Given that there was significant difference in HPV vaccine completion rates between sexual minority and heterosexual males, sexual minority males' completion rates appeared higher (38.2%, 95% CI: 23.0–53.3% vs. 17.0%, 95% CI: 0.0–36.9%; *p*=0.14; Table 2), though not statistically significant.

**Table 2.** Human Papillomavirus Vaccination Completion (Three Doses) Rates by Sexual Orientation in National Health and Nutrition Examination Survey (2007–2016)

Factors	Overall (***n***=687)	Heterosexuals^a,b^ (***n***=601)	Sexual minorities^a,c^ (***n***=86)	***p*** ^d^
	% (95% CI)	% (95% CI)	% (95% CI)	
HPV vaccination completion rate	56.9 (51.0–62.8)	56.8 (50.8–62.7)	57.7 (45.3–70.0)	0.88
Age group				
26 years and younger	52.9 (46.5–59.3)	52.6 (46.1–59.2)	54.6 (41.3–68.0)	0.76
Older than 26 years	67.8 (58.9–76.8)	67.6 (58.7–76.5)	69.8 (43.0–96.7)	0.87
Gender^e^				
Female	61.4 (56.0–66.7)	61.0 (55.4–66.6)	63.7 (51.9–75.5)	0.66
Male	36.1 (22.1–50.2)	38.2 (23.0–53.3)	17.0 (0.0–36.9)	0.14
Race				
White	60.4 (52.1–68.6)	60.1 (51.7–68.5)	62.1 (44.7–79.6)	0.81
Black	46.4 (37.1–55.7)	45.9 (37.0–54.8)	49.6 (28.9–70.3)	0.69
Hispanic	53.1 (44.0–62.1)	53.3 (43.3–63.2)	51.3 (27.1–75.6)	0.89
Other^f^	51.5 (40.7–62.2)	52.7 (41.5–63.9)	44.7 (16.7–72.6)	0.57
Education				
Below high school	41.9 (25.2–58.7)	46.0 (29.1–62.8)	12.6 (0.0–37.2)	0.06
High school	53.5 (42.2–64.8)	54.9 (41.9–67.9)	47.1 (26.4–67.8)	0.54
Associates and some college	55.5 (48.7–62.3)	54.5 (47.4–61.5)	621 (44.1–80.0)	0.43
College graduate and above	63.4 (53.5–73.2)	63.1 (52.8–73.4)	65.7 (42.6–88.8)	0.83
Income				
High	67.3 (58.1–76.5)	67.2 (58.4–76.0)	68.1 (43.4–92.8)	0.94
Middle	55.4 (46.7–64.0)	55.9 (46.4–65.4)	51.8 (33.0–70.6)	0.70
Low	47.6 (40.3–54.9)	46.3 (38.4–54.2)	54.8 (33.2–76.4)	0.45
Type of insurance				
None	44.0 (34.8–53.2)	44.4 (34.4–54.4)	41.5 (17.4–65.6)	0.82
Private	60.7 (53.2–68.3)	61.0 (53.5–68.5)	58.9 (40.8–76.9)	0.81
Medicare	0	0	0	
Medicaid	57.0 (46.1–67.9)	55.7 (44.2–67.1)	72.0 (45.3–98.7)	0.29
Other^g^	53.2 (36.5–69.9)	50.4 (31.8–68.9)	69.8 (36.0–100.0)	0.37
Setting of routine health care received				
No place	52.9 (42.3–63.5)	50.6 (39.2–61.9)	64.7 (41.6–87.9)	0.29
Clinic or health center	42.8 (32.5–53.1)	44.7 (33.0–56.4)	32.2 (14.6–49.8)	0.30
Doctor's office or HMO	66.7 (59.3–74.2)	66.6 (59.2–74.0)	67.9 (52.5–83.3)	0.86
Hospital emergency room	22.3 (7.8–36.7)	22.4 (6.9–37.9)	21.3 (0.0–60.8)	0.96
Other^h^	44.8 (25.8–63.7)	39.1 (19.6–58.7)	72.1 (25.6–100.0)	0.21
Time period^*^				
2007–2008	33.1 (28.2–38.1)	34.8 (29.5–40.0)	0.0 (0.0–0.0)	
2009–2010	52.4 (39.1–65.7)	54.3 (40.7–68.0)	32.7 (0.4–65.1)	0.25
2011–2012	59.7 (45.5–74.0)	57.1 (43.9–70.3)	75.2 (40.1–100.0)	0.09
2013–2014	60.0 (49.2–70.9)	60.9 (50.5–71.3)	54.7 (31.8–77.6)	0.53
2015–2016	56.9 (46.4–67.5)	57.0 (45.5–68.6)	56.4 (37.6–75.2)	0.95

*Note:* Numbers do not add up to 100% because of subgroup analyses. Data report complete human papillomavirus vaccination rate (three doses) out of those who initiated the vaccine.

^*^ Overall: *p*-trend <0.001; heterosexuals: *p*-trend=0.004; sexual minority: *p*-trend=0.001.

^a^ Participants were asked in the survey, “Do you think of yourself as…” with options given as heterosexual or straight, “homosexual, bisexual, something else, not sure, refused, and don't know” (https://www.cdc.gov/nchs/nhanes).

^b^ Male heterosexuals=111; female heterosexuals=490.

^c^ Male homosexuals=8; male bisexuals=3; female homosexuals=6; female bisexuals=69.

^d^ Chi-square test used to test for association between sexual behavior and HPV vaccination by each category.

^e^ Gender options in NHANES were presented as female or male only.

^f^ Other race includes multirace and any other race not specified in the NHANES database.

^g^ Other insurance includes Medi-Gap, State Children's Health Insurance Program, military health insurance, Indian health service, state-sponsored health plan, and other government insurance.

^h^ Other setting of health care includes hospital outpatient department and other place not specified in the NHANES database.

Factors that predicted initiation of vaccination included female gender (odds ratio [OR]=3.29, 95% CI: 2.38–4.54; *p*<0.001), younger age, ≤26 years, (OR=3.18, 95% CI: 2.56–3.94; *p*<0.001), receiving their health care from the doctor's office or HMO (OR=1.38, 95% CI: 1.05–1.82; *p*=0.02), and sexual minority status (OR=1.73, 95% CI: 1.20–2.49; *p*=0.004; Table 3). In addition, we conducted another multivariable regression analysis to examine the interaction terms of sexual minority with gender as well as with race; however, the results showed null findings.

**Table 3.** Multivariate Logistic Regression in Predicting Human Papillomavirus Vaccination Initiation (≥1 Dose)

Factors	OR (95% CI)	***p***
Sexual orientation		
Heterosexuals	1	
Sexual minority	1.73 (1.20–2.49)	**0.004**
Age group		
>26 years	1	
26 years and younger	3.18 (2.56–3.94)	**<0.001**
Gender^a^		
Male	1	
Female	3.29 (2.38–4.54)	**<0.001**
Race		
White	1	
Black	1.03 (0.79–1.34)	0.84
Hispanic	0.78 (0.60–1.02)	0.07
Other^b^	0.78 (0.56–1.08)	0.14
Type of insurance		
None	1	
Private	1.68 (1.27–2.23)	**<0.001**
Medicare	0.70 (0.12–4.07)	0.69
Medicaid	1.65 (1.13–2.40)	**0.01**
Other^c^	2.09 (1.35–3.22)	**0.001**
Education		
Below high school	1	
High school	1.73 (1.12–2.65)	**0.01**
Associates and some college	2.77 (1.93–3.99)	**<0.001**
College graduate and above	2.60 (1.60–4.22)	**<0.001**
Income		
Low	1	
Middle	1.26 (0.95–1.66)	0.11
High	1.13 (0.80–1.59)	0.49
Health routine		
No place	1	
Clinic or health center	1.25 (0.90–1.72)	0.18
Doctor's office or HMO	1.38 (1.05–1.82)	**0.02**
Hospital emergency room	1.46 (0.84–2.55)	0.18
Other^d^	2.69 (1.67–4.34)	**<0.001**

Boldface indicates statistical significance (*p*<0.05).

^a^ Gender options in NHANES are presented as female or male only.

^b^ Other race includes multirace and any other race not specified in the NHANES database.

^c^ Other insurance includes Medi-Gap, State Children's Health Insurance Program, military health insurance, Indian health service, state-sponsored health plan, and other government insurance.

^d^ Other setting of health care includes hospital outpatient department and other place not specified in the NHANES database.

OR=odds ratio.

## Discussion

The overall rate of vaccination initiation was lower in heterosexual adults than in sexual minority adults. Furthermore, given the lack of significant difference in HPV vaccine completion rates and overlapping CIs, there is no statistically significant difference detected between sexual minority and heterosexual males. Sexual minority males' completion rates appeared to be higher, though this was not statistically significant. We speculate that sexual minority males obtain initial access to HPV vaccination in health care centers but these settings may not have a system of care provision that facilitates continuity of care to complete the vaccination.^7^

However, others have shown that community health centers provide better continuity of care than hospital outpatient departments and physician offices.^8^ Another potential barrier to completing the vaccination series may be that sexual minority males have initiated care but experienced discrimination from health care providers that discouraged ongoing and follow-up care.^9^

To further understand this difference in vaccination rates in heterosexual versus sexual minority adults, we identified the locations of access to health care. Of those vaccinated, the sexual minorities responded that they received more routine care in clinical health care centers and medical doctor's offices where the HPV vaccination rate is relatively high in comparison with heterosexuals. A prior study showed that pediatricians are more likely to recommend vaccination than family medicine doctors.^10^ Better access to health care, particularly one that is tailored to an individual's sexual orientation, can potentially overcome barriers to receiving HPV vaccine.

Our study was likely limited by recall bias as NHANES relies on participants' self-reports of HPV vaccination and their routine health care setting. We also restricted our data set to 20–36 years because sexual information data were only available for those ≥20 years.

Concerning the classification of sexual orientation, we were unable to account for heterogeneity in sexual minority subgroups including other sexual orientations (e.g., asexual, pansexual, queer, two-spirit) or sexual fluidity. Given NHANES have no assessment of gender as distinct from sex assigned at birth, we were unable to examine gender identity across the gender spectrum.^11^ However, this is one of the few studies to use NHANES data addressing HPV vaccination among sexual minorities and examining trends in vaccination over time.

Overall, heterosexuals had lower rates of HPV vaccination than sexual minorities, vaccination completion rates were lower in the sexual minority males. Understanding barriers and designing interventions to enhance completion are critical to improving the health care status of sexual minorities.^3^ Large databases with robust representation of sexual minorities may facilitate understanding of primary prevention efforts.

## Abbreviations Used

CI: confidence interval
HPV: human papillomavirus
NHANES: National Health and Nutrition Examination Survey
OR: odds ratio
